# Grain Amaranth Is Associated with Improved Hepatic and Renal Calcium Metabolism in Type 2 Diabetes Mellitus of Male Wistar Rats

**DOI:** 10.1155/2018/4098942

**Published:** 2018-10-18

**Authors:** Keneth Iceland Kasozi, Sarah Namubiru, Abass Alao Safiriyu, Herbert Izo Ninsiima, Dorothy Nakimbugwe, Monica Namayanja, Miriela Betancourt Valladares

**Affiliations:** ^1^Department of Physiology, Faculty of Biomedical Sciences, Kampala International University Western Campus, Box 71, Bushenyi, Uganda; ^2^College of Veterinary Medicine Animal Resources and Biosecurity, Makerere University, Box 7062, Kampala, Uganda; ^3^Department of Anatomy and Physiology, School of Medicine, Kabale University, Uganda; ^4^Department of Food Technology & Nutrition, School of Food Technology, Nutrition & Bio-Engineering, Makerere University, Box 7062, Kampala, Uganda; ^5^Department of Biochemistry, Faculty of Biomedical Sciences, Kampala International University Western Campus, Box 71, Bushenyi, Uganda; ^6^Department of Physiology, Faculty of Medicine, Mbarara University of Science and Technology, Box 1410 Mbarara, Uganda; ^7^Department of Biomedical Sciences, Faculty of Dentistry, Camaguey Medical University, Cuba

## Abstract

**Background:**

Dysregulation of calcium signaling is a hallmark of diabetes mellitus (DM) and grain amaranth (AG) has antidiabetic properties. Information on the mechanism of action of AG on blood, renal, and hepatic tissues is sparse, although it continues to be an important alternative medicinal plant in several developing countries. The objective of the study was to determine key changes in calcium levels and s100a1 protein levels and antioxidant and histopathologic changes in blood, renal, and hepatic tissues of male diabetic Wistar rats.

**Materials and Methods:**

This was an experimental study in which 30 male Wistar rats were kept for 5 weeks (6 groups, N =5). Groups 1-IV had T2DM induced using Nicotinamide and Streptozotocin: Group I, Mixtard®; group II, positive control; group III, 25% AG; group IV, 50% AG. Furthermore, group V consisted of normal rats given 50% GA and group VI was negative control. Blood, renal, and hepatic tissues were collected and analyzed for calcium, s100a1 protein levels, and antioxidant and histopathological changes.

**Results and Discussion:**

In blood, renal, and hepatic tissue, calcium and s100a1 levels were low during T2DM and these increased following AG supplementation. This was important for improved metabolic processes, thus leading to the low malondialdehyde (MDA) and glutathione peroxidase (GPx) activity in the tissues. Efficient antioxidant status was important for improved calcium signaling mechanisms, thus leading to improved tissue function and protection demonstrating the importance of AG as an alternative medicinal source through the calcium signaling pathway.

**Conclusion:**

Grain amaranth exerts its antidiabetic properties through improved calcium homeostasis in blood, kidney, and liver.

## 1. Background

Calcium (Ca^2+^) is an essential mineral of eukaryotic cells [1], since it is a versatile intracellular signaler that controls various cellular functions; therefore, Ca^2+^ signals need to be flexible yet precisely regulated [2] as it has been shown to affect key physiological processes in several body tissues [3]. This is intriguing since 1% of the total body calcium is used for metabolic processes such as blood clotting, muscle contraction, and nerve transmission, showing its relevance in blood, smooth muscles, and visceral organs [3]. Besides controlling gene transcription and growth, Ca^2+^ regulates the contraction and relaxation in muscle tissue [4]. Ca^2+^ cycling in muscle tissue is regulated by a plethora of proteins, transmitting the Ca^2+^ messages with precision and in a temporally and spatially coordinated manner. One of the specific Ca^2+^ binding protein families within muscle cells is the S100 protein family which plays a critical role in the excitation mechanism [5]. An inability of the tissues to regulate calcium levels has been associated with the development of type 2 diabetes mellitus (T2DM) due to defects in both insulin secretion and action, thus leading to Ca^2+^ dyshomeostasis [6] and oxidative stress (OS) in the tissues [3, 7–9]; however, little is known about the mechanism of action of several ethnomedical plants used in the management of T2DM in several developing countries.

In the management of T2DM, vegetables such as grain amaranth (AG) have been used [10, 11]. GA (*Amaranthus hypochondriacus*) which is a highly nutritious plant [12] has extensively been promoted in several developing countries especially in Africa [13, 14]. Phytochemical analysis of AG has shown that it is rich in vitamin C, *β*-carotene, folic acid, iron, calcium, proteins, phosphorus, iron, potassium, zinc, vitamin E, vitamin *β*-complex, riboflavin, amino acids, and thiamin [10, 15] and these have established properties related to protection against oxidation, control of serum lipid levels, and decrease inflammation [16]. In particular AG has been shown to have about 159 mg of calcium per 100 g of grain, thus serving as a crucial calcium supplementary source to man [10, 17]. In addition AG has demonstrated hypoglycemic and hypercholesterol properties [18], and it has been shown to control weight gain due to its high calcium content [19–22] demonstrating its importance in DM. In several studies using GA, a lot of emphasis has been placed on the use of leaves for their antioxidant activity [23]; however a study by Muyonga et al. [24] showed that processed seeds have a superior antioxidant activity than leaves [24–26]. These observations imply that processed AG would be an ideal ethnomedicinal plant for use in the management of T2DM in humans, showing a need to generate evidence for its mechanism of action.

In body tissues, total serum calcium is a direct measure of parathyroid activity and a positive relationship has been demonstrated to exist between serum calcium and T2DM in humans showing that altered calcium homeostasis is central in the pathophysiology of T2DM [27, 28]. The ability of AG to control blood glucose imbalance has also been investigated in previous studies [29]; however, information on changes in blood calcium levels and s100a1 levels under differential grain amaranth feed supplementation is sparsely available. In the liver, calcium is important for the regulation of subcellular and cellular physiological processes such as bile secretion, glucose metabolism, cell proliferation, and apoptosis [30]. The liver plays a crucial role in the maintenance of normal glucose levels through its activation of glycogenolysis and gluconeogenesis under the actions of glucagon and insulin, respectively [31]. These metabolic processes are dependent on calcium levels which have to be maintained within the normal ranges for efficient mitochondrial function [32]. In DM, calcium deregulation leads to initiation of apoptosis and development of necrotic lesions [30, 33–35], showing that key metabolic functions in the liver would be affected. In failing hepatocytes, the s100al proteins have been shown to play a crucial role in tissue rescue [36], showing a need to identify basic changes in s100a1 expression in the liver following AG supplementation in DM.

In the kidney, complexed and ionized calcium together are termed as the ultra-filterable calcium and are freely filtered by the glomerulus [37]. In the proximal convoluted tubule (PCT), approximately 70% of filtered calcium is reabsorbed through facilitated diffusion, thus showing the role of active transport mechanisms. Furthermore, the epithelial cells in the loop of Henle (LoH) have calcium transport proteins which respond to actions of PTH and vitamin D3; however, these calcium transporters have been found to be downregulated during DM [37–39]. This leads to decreased calcium reabsorption in the renal tissue leading to increased calcium in urine that results in grave prognosis [40]. Increased calcium dysregulation in the kidney leads to disruption of metabolic processes in the renal mitochondria leading to reduced ATP production and increased production of reactive oxygen species (ROS). These subsequently lead to increased tissue damage in the kidney and development of renal insufficiency [41, 42]. The s100a1 proteins have been shown to be of clinical diagnostic importance in renal disease [36, 43, 44]. This justified the need to assess s100a1 protein levels in the kidney during DM following AG supplementary feeding. This was important since AG has been shown to possess hepato-protective properties [45]; however, information on its mechanism of action through calcium metabolism is sparse. Furthermore, the kidney plays a crucial role in calcium homeostasis [37–39], and yet GA has significantly high calcium concentrations, i.e., 159 mg per 100 g of grain [10, 17]. The objective of the study was to determine levels of blood calcium concentrations, s100a1 protein, antioxidant levels, and tissue changes following grain amaranth feed supplementation in T2DM male Wistar rats.

## 2. Materials and Methods

### 2.1. Study Design

This was an experimental study in which quantitative and qualitative data were collected from 2-month-old T2DM male Wistar rats. The study consisted of six study groups each consisting of 5 male Wistar rats. Rats in groups I –IV were diabetic. Group I was treated with Mixtard® [46]. Group II was the positive control (not treated); Groups III and IV were on 25% and 50% w/w grain amaranth supplementation, respectively. In addition, groups V and VI consisted of normal rats and these were given 50% AG and control feed (negative control), respectively, and the rats were kept for a total of 5 weeks.

### 2.2. Induction of T2DM in Male Wistar Rats

The research model was a Nicotinamide and Streptozotocin (STZ) model of T2DM [47]. After acclimatization, 30 adults (age = 8 wks) were used. T2DM was induced (n = 20 rats) using STZ (60 mg/ml) and Nicotinamide (120 mg/kg) intraperitoneal as described previously [48, 49]. Rats with hyperglycemia (≥250 mg/dl) were used for the purpose of this study as previously described [49, 50].

### 2.3. Grain Amaranth Processing

Commercial* Amaranthus hypochondriacus* was got from the Department of Food Technology and Human Nutrition, College of Agricultural and Environmental Sciences, Makerere University. This was processed to create popped grain amaranth by heating at 260°C for 15 seconds [51] for increased nutrient bioavailability [24]. Two feeds were formulated by supplementing regular rat pellet with popped amaranth, i.e., low and high supplementation at 25% w/w and 50% w/w, respectively. Water was added to the mixture to moisten it and pellets were formed and dried at 105°C for 24 hrs in an oven (WTE Binder, type 19240300002000, no. 950228, Germany) for preservation and stored in a sac at room temperature.

### 2.4. Laboratory Analysis

At the end of 5 weeks, animals were euthanized using sodium pentobarbitone injected intraperitoneally since this is acceptable in rodents [52]. The blood, kidney, and liver were harvested from each rat and placed in sterile sample bottles. Samples were divided into duplicates for biochemical and histological analysis. Samples for biochemical analysis were subsequently homogenized in 1M phosphate buffer saline. These were then centrifuged at 3000 rpm for 5 min and the filtrate was collected into sterile eppendorf tubes which were stored in a refrigerator at -20°C for biochemical analysis. Samples for histological analysis were placed in 10% buffered formalin.

#### 2.4.1. Determination of Tissue Calcium

Calcium concentrations in the experimental diets and the tissues were determined using an atomic absorbance spectrometry (AAS) [53]. The AAS (Perkin-Elmer, model GBC932AA, USA) was set up according to manufacturer's recommendations and an equation from the standard curve (absorbance = 450 nm) was used to determine calcium concentrations for each sample.

#### 2.4.2. Determination of s100a1 Protein Levels

This was done by using ELISA standard protocol [54]. The protein s100a1 was determined using a commercial test kit (Santa Cruz, Biotechnology, USA, Texas) in accord with manufacturer's recommendations. The s100a1 variant used in this study was catalogue SC-71992 with a Gene ID of 6271 (1q21.3) in humans and that of 20193 (3F1) in rats. The optical density was measured at 450 nm for the s100a1 proteins [55] using an automatic ELISA plate reader (Biotech, USA).

#### 2.4.3. Determination of Oxidative and Antioxidant Activity

This was done by using malondialdehyde (MDA) and glutathione peroxidase (GPx) as markers for oxidative and antioxidant activity. Determination of MDA was done by the nonspecific Thiobarbituric acid reactive substances (TBARS) measurement. This assay is based on reaction of a chromogenic reagent, 2-thiobarbituric acid with MDA [56]. In brief 1M of MDA reacts with 2M of 2-thiobarbituric acid (TBA) to yield a chromophore and the absorbance was taken at 540 nm using trichloro acetate, TBA, hydrochloric acid, and sodium hydroxide [57].

Glutathione peroxidase (GPx) activity was determined and measured using the method of Yutaka [58] following the formation of GSSG using a coupled enzyme system with glutathione reductase (GRx in *µ*M NADPH oxidized mg^−1^ protein min^−1^) as shown in Table 1. This was important since the formation of glutathione (GSSG) is catalyzed by GPx coupled by the recycling of GSSH back to GSH using GSSG-R (glutathione reductase). NADPH is oxidized to NADP^+^. The change in absorbance due to NADPH oxidation was monitored and was indicative of GPx activity [59].

After making the reaction volumes, the mixture was vortexed at room temperature, incubated at 37°C for 15 minutes in a water bath. The activity of the samples was enhanced by adding 5% TCA. The samples were then centrifuged at 3000 rpm for 5 minutes. The supernatant was collected and transferred into 96-well plates and an ELISA plate reader was used as described previously [58].

#### 2.4.4. Histopathology Determination

Sections of liver and kidney tissue blocks of each rat were analyzed according to asystematic random embedding, random sectioning, and sampling method [60]. Microscopic changes were assessed using a light microscopy and described descriptively.

### 2.5. Data Analysis

Quantitative data was analyzed using Graph Pad Prism Version 6. Information was presented as mean ± SD, a Tukey's test was conducted to determine sources of variation, and significant differences were reported when P < 0.05. Data from histological analysis was summarized and presented in paragraphs.

## 3. Results

### 3.1. Feed Calcium Levels and Changes in Weight during the Experimental Period in Male Wistar Rats

**Figure 1:**
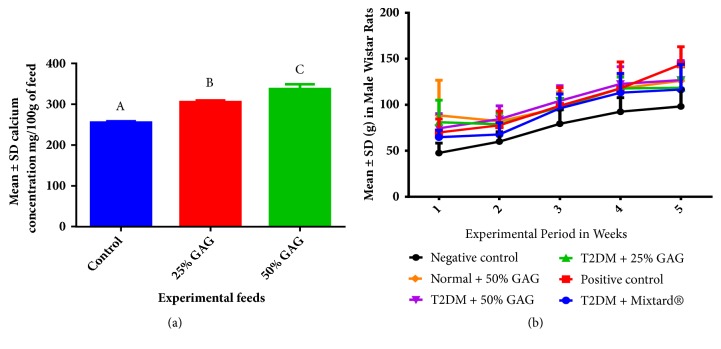
Calcium concentration in feeds and growth rate. (a) Feed calcium concentrations. (b) Weights of experimental animals.

Mean calcium concentrations in experimental diets were highest in the order of AG 50% > AG 25% > control feed as shown in Figure 1(a). In addition, weights were generally higher in the positive control as compared to other experimental groups, although these were not significantly different (Table 3). Generally, the growth rate followed the general growth sigmoid curve as shown in Figure 1(b).

### 3.2. Calcium Concentrations in Blood, Kidney, and Liver in Male Wistar Rats

Generally, calcium concentrations were highest in the order of blood > kidney > liver. The concentration (mean ± SD) of blood calcium found in the T2DM rats was 11.27 ± 0.52 mg/dl and 12.15 ± 0.84 mg/dl under 25% AG and 50% AG supplementation, respectively (Table 2). These concentrations were both lower than those measured in the negative control which had 12.65 ± 0.86 mg/dl. Significant differences (ANOVA,* P< 0.05*) were found to exist mainly in the positive control and 25% AG experimental groups (Table 3). In addition, calcium concentrations were higher in the kidney than the liver. In the kidney, calcium concentrations were found to be 0.53 ± 0.18 mg/dl and 0.39 ± 0.17 mg/dl in 25% and 50% AG supplement, respectively (Table 2). Significant differences in calcium concentrations were found to be mainly in the positive control (Table 3), showing that AG improved on calcium levels in the kidney during DM. Significant differences were found to exist in between the experimental groups (ANOVA,* P = 0.0065*) in which low concentrations of calcium were shown to be associated with only the positive control (T2DM without treatment), while AG was associated with increased calcium concentrations relative to the positive control (Table 3). In the liver, calcium levels were also lowest in the positive control, although no significant differences were found between the experimental groups (ANOVA, P > 0.05).

### 3.3. Changes in s100a1 Protein Levels in Blood, Kidney, and Liver in Male Wistar Rats

**Figure 2:**
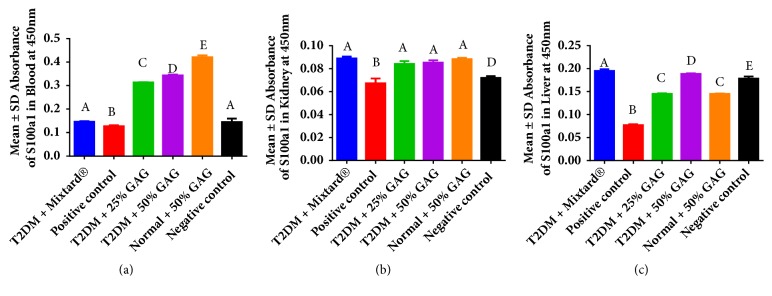
S100a1 protein levels in blood, kidney, and liver following grain amaranth feed supplementation in T2DM male Wistar rats. Image (a) blood, (b) kidney, and (c) liver. In blood, grain amaranth at 25% and 50% led to an increase of s100a1 protein levels during T2DM. In the kidney there were no dose related changes following AG supplementation in the s100a1 protein levels. In the liver, there were dose related changes in s100a1 protein levels following AG supplementation to T2DM male Wistar rats. Observations in the study show that AG supplementation has strong effects in the order of blood > liver > kidney.

In blood, kidney, and liver, s100a1 levels were low in the positive control and these increased significantly (P < 0.05) following AG feed supplementation (Table 3). In blood and liver, increase in s100a1 protein levels was dose dependent (Figures 2(a) and 2(c)) while in the kidney, this was dose independent (Figure 2(b)). Furthermore, s100a1 levels were generally high in the order of blood > liver > kidney as shown in Figure 2.

### 3.4. Levels of Oxidant and Antioxidant Activity in Blood, Kidney, and Liver

**Figure 3:**
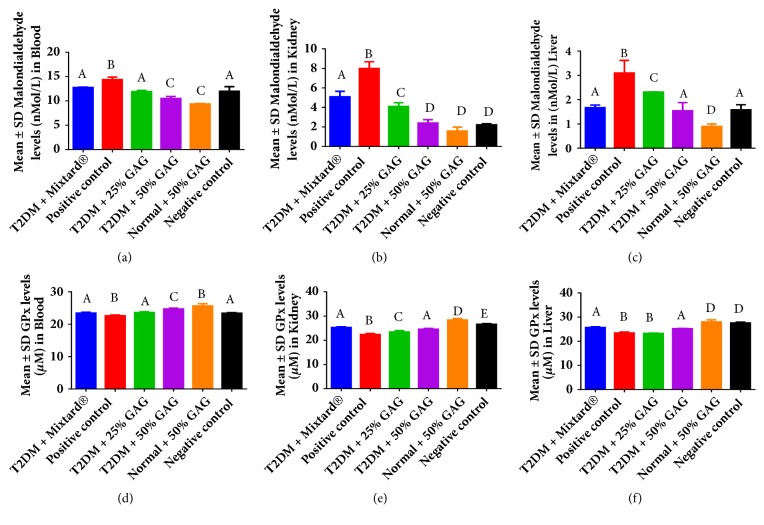
Levels of oxidants and antioxidants in blood, kidney, and liver of male Wistar rats after grain amaranth feeding. Images (a), (b), (c) are malondialdehyde levels while images (d), (e), (f) are glutathione peroxidase levels for blood, kidney, and liver, respectively.

MDA levels were elevated in the positive control in all the tissues. These subsequently reduced (*P < 0.05*) in a dose dependent manner in all the tissues following grain amaranth supplementation. Glutathione was a key antioxidant manner measured in the tissues and the study showed that glutathione reductase (GPx) levels are lowest in the positive control and significantly elevated (*P < 0.05*) in a dose dependent manner following grain amaranth supplementation in blood and kidney (Table 3). In the liver, grain amaranth supplementation at 25% had no significant differences from the positive control on GPx levels (*P > 0.05*) as shown in Figure 3.

### 3.5. Major Changes in Histological Structure of the Kidney and Liver Tissues

**Figure 4:**
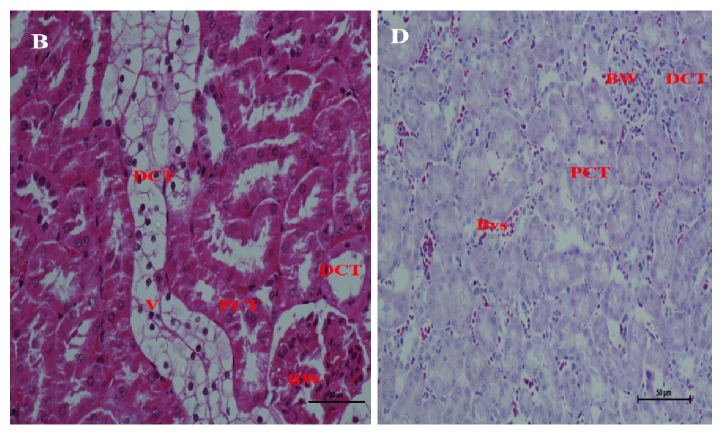
Changes in renal tissue following AG supplementation in male diabetic rats.** KEY: **Image B: positive control, D: negative control. BW: Bowman's capsule, PCT: proximal convoluted tubule; DCT: distal convoluted tubule; Bvs: blood vessels; V: vacuolation. Tubule degeneration in T2DM without treatment, AG: grain amaranth.

Renal tubular vacuolation in the positive control was predominant in the PCT and DCT. No significant changes were seen in the negative control and AG supplementation as shown in Figure 4.

**Figure 5:**
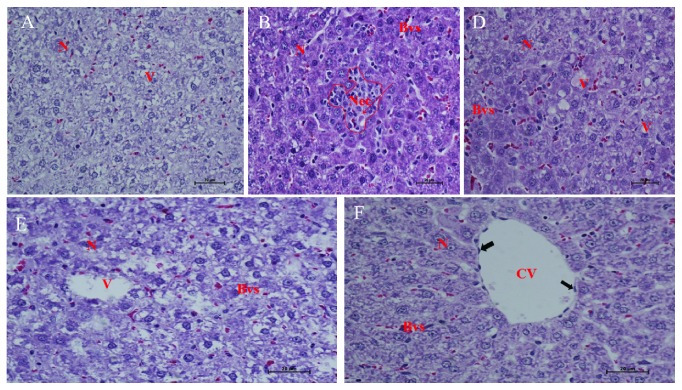
Structural changes in hepatic tissue after grain amaranth supplementation in male diabetic Wistar rats.** KEY:** Image A: T2DM + Mixtard®, B: positive control; D: T2DM + 50% AG; E: normal + 50% AG; F: negative control. N: nuclei of hepatocyte, Bvs: blood vessel, V: vacuolation, CV: central vein, black arrows: endothelium of blood vessel, Nec: necrosis. Mild vacuolation seen in T2DM and AG supplementation groups, but severe vacuolation and necrosis in T2DM without treatment. No changes in normal group.

Furthermore, absolute multiple vacuolation, necrosis, and mononuclear cell infiltration were found in hepatic tissue of the positive control, while AG supplementation led to mild vacuolation. There were no significant differences in vacuolation observed in the AG supplemented and negative control as shown in Figure 5.

## 4. Discussion

The mean body weight was highest in the positive control (Figure 1(b)), demonstrating the importance of monitoring weight gain in diabetes [21]. Grain amaranth (AG) supplementation was associated with controlled weight gain in diabetic rats demonstrating the protective effect offered by grain amaranth in controlling weight gain [22]. This was because AG at higher concentrations had elevated calcium concentrations (Figure 1(a)), which led to low weights in diabetic rats under AG. This was in agreement with previous findings in which high calcium diets have been associated with weight loss [19, 20].

Calcium concentrations in the tissues were generally in the order of blood > kidney > liver. This was important since blood is a media for transport of calcium to various body tissues under direct action of parathyroid gland [27, 28]. Observations in the study show that AG improves on calcium homeostasis during DM (Table 2), thus being of protective effect on several body organs which depend on calcium to perform their functions [9]. This was important since calcium is an important secondary messenger whose levels within the body have to be adequately regulated [61]. In the kidney, AG led to increased calcium concentration in the renal tissues, probably through increased calcium transporter abundance [39]. Improved calcium homeostasis during AG supplementation led to increased s100al calcium handling proteins (Figure 2(b)), which led to efficient renal reabsorption of calcium into the tissues, leading to a better prognosis in DM than in the positive control [40]. Bearing in mind that the s100a1 proteins are expressed in a majority of the tissues, the study showed that these are mainly expressed in the order of blood > liver > kidney under AG supplementation (Figure 2). These findings are in agreement with previous findings in which s100al proteins have been shown to be crucial markers for renal and hepatic function [36, 44].

Increased calcium transport through increased transcription of the s100al proteins in diabetic tissues following AG supplementation would lead to efficient mitochondrial function and production of enough ATP to activate several calcium transporters within the cytosol [4, 5]. These transporters would be deactivated by an excessive buildup of reactive oxygen species; however, AG supplementation was associated with a low MDA and high GPx activity (Figure 3). Bearing in mind that the antioxidant activity of AG has been investigated extensively in previous studies, findings in this study show that s100a1 protein levels in DM are synergistic to the increased antioxidant activity of AG. This synergistic role would also imply that the increased presence of antioxidants helps to shield cytosolic proteins (including the s100a1 proteins) against the severe effects of oxidative stress associated with DM, since the production of reactive oxygen species is minimized through efficient energy metabolic processes in the mitochondrion [6–8]. Furthermore, higher AG supplementation led to significantly higher GPx levels in both blood and kidney (Table 3), probably due to the direct activity of parathyroid hormone in these tissues [27, 28, 37–39].

Grain amaranth had an increased antioxidant activity which offered protection to the tissues, leading to minimal pathological lesions in blood, kidney, and liver tissues of DM rats (Figures 4 and 5) and this was in agreement with previous studies [24–26]. Necrotic lesions were only significantly observed in the positive control which was in agreement with previous studies that DM leads to tissue damage due to accumulation of reactive oxygen species which predispose cell membranes to lipid peroxidation [41, 42]. Improved renal and hepatic calcium homeostasis through increased s100a1 protein levels inevitably led to increased tissue protection in DM. Observations from the study reemphasize the need to continuously use calcium as a diagnostic marker for monitoring DM patients in humans and promote the consumption of grain amaranth as a food supplement in alternative medicinal practices for several developing countries. The study demonstrates the importance of the metabolic pathway in the assimilation of grain amaranth, and this would be reemphasized by prospective studies on AG. This would be important in prospective toxicological and drug development studies since the breakdown of Nicotinamide and Streptozotocin may be associated with primary xenobiotic metabolites which would affect the outcomes observed in this study.

## 5. Conclusion

Grain amaranth supplementation increased content of Ca in the diet and improved calcium signaling in blood, kidney, and liver of diabetic rats. This occurs through increased expression of the s100a1 calcium transport proteins which lead to improved calcium homeostasis in the tissues. Increased s100a1 protein levels are also favored by the strong antioxidant activity of grain amaranth leading to increased tissue protection against oxidative stress which is common in DM. This study offers evidence on the mechanism of action of grain amaranth in the management of T2DM in humans as it is commonly used in several developing countries. More information, however, still remains to be established on the role played by other second messengers in the presence of grain amaranth, as this would offer a holistic picture on the synergistic and antagonistic role played by different pathway mechanisms. In addition, authors placed emphasis on calcium content in grain amaranth; however, effects of oxalates and exogenous calcium and other chemical constituents under feed supplementation would offer added information which would guide prospective clinical studies.

## Figures and Tables

**Table 1:** Materials on GPx determination.

Reagents (*µ*l)	Blank	Reference standard	Sample
Phosphate buffer	400	300	150
GSH	0	100	100
Sodium Azide	50	50	50
Hydrogen peroxide	50	50	50
Supernatant	0	0	150
Total	500	500	500

**Table 2:** Mean calcium concentrations in selected tissues following GAG supplementation in T2DM.

Experimental Group	N	Blood	Kidney	Liver
		Calcium concentrations (Mean ± SD) mg/dl		
T2DM + Mixtard® 0.5 mg/kg	5	12.83 ± 0.96	0.37 ± 0.14	0.20 ± 0.16
Positive control	5	10.75 ± 0.62^*∗∗*^	0.11 ± 0.07	0.06 ± 0.04
T2DM + 25% GA	5	11.27 ± 0.52^*∗*^	0.53 ± 0.18^*∗∗*^	0.37 ± 0.21
T2DM + 50% GAG feed supplement	5	12.15 ± 0.84	0.39 ± 0.17	0.28 ± 0.20
Normal rats + 50% GAG feed supplement	5	11.85 ± 0.71	0.36 ± 0.19	0.14 ± 0.13
Negative control	5	12.65 ± 0.86	0.55 ± 0.14	0.34 ± 0.19

**KEY:** In-group comparisons (ANOVA; *P < 0.05*) and multiple Tukey's test. In blood, ^*∗∗*^P < 0.01, ^*∗*^P < 0.05. T2DM: type 2 diabetes mellitus; GAG: grain amaranth grain; N: number of rats.

**Table 3:** P values on Tukey's tests for weights, calcium, s100al protein levels, MDA, and GPx in experimental groups.

Multiple Tukey's test comparisons	N	Weight	Calcium			S100a1			MDA			GPx		
			Blood	Renal	Liver	Blood	Renal	Liver	Blood	Renal	Liver	Blood	Renal	Liver
T2DM + Mixtard® vs. Positive control	5	0.9826	0.0049	0.2413	0.6827	0.0051	< 0.0001	< 0.0001	0.0027	< 0.0001	< 0.0001	0.0209	< 0.0001	< 0.0001
T2DM + Mixtard® vs. T2DM + 25% GAG	5	0.9963	0.0255	0.6092	0.0829	< 0.0001	0.0389	< 0.0001	0.2173	0.0293	0.0467	0.9722	< 0.0001	< 0.0001
T2DM + Mixtard® vs. T2DM + 50% GAG	5	0.9746	0.6888	0.9999	0.3361	< 0.0001	0.194	0.0079	< 0.0001	< 0.0001	0.9858	0.0007	0.1609	0.7177
T2DM + Mixtard® vs. Normal + 50% GAG	5	0.9772	0.3104	> 0.9999	0.9717	< 0.0001	0.9985	< 0.0001	< 0.0001	< 0.0001	0.0039	< 0.0001	< 0.0001	< 0.0001
T2DM + Mixtard® vs. Negative control	5	0.8763	0.9988	0.5177	0.1284	0.9996	< 0.0001	< 0.0001	0.2601	< 0.0001	0.9953	0.9995	0.0007	0.0005
Positive control vs. T2DM + 25% GAG	5	> 0.9999	0.9	0.0072	0.9533	< 0.0001	< 0.0001	< 0.0001	< 0.0001	< 0.0001	0.0118	0.0031	0.0118	0.9622
Positive control vs. T2DM + 50% GAG	5	> 0.9999	0.0849	0.1273	0.2829	< 0.0001	< 0.0001	< 0.0001	< 0.0001	< 0.0001	< 0.0001	< 0.0001	< 0.0001	0.0003
Positive control vs. Normal + 50% GAG	5	> 0.9999	0.2667	0.2706	0.9999	< 0.0001	< 0.0001	< 0.0001	< 0.0001	< 0.0001	< 0.0001	< 0.0001	< 0.0001	< 0.0001
Positive control vs. Negative control	5	0.4961	0.0081	0.0051	0.7526	0.0105	0.052	< 0.0001	< 0.0001	< 0.0001	< 0.0001	0.04	< 0.0001	< 0.0001
T2DM + 25% GAG vs. T2DM + 50% GAG	5	0.9998	0.3751	0.7045	0.9888	< 0.0001	0.9644	< 0.0001	0.0036	0.0004	0.0129	0.0022	0.0136	< 0.0001
T2DM + 25% GAG vs. Normal + 50% GAG	5	0.9999	0.7792	0.5632	0.3998	< 0.0001	0.0908	> 0.9999	< 0.0001	< 0.0001	< 0.0001	< 0.0001	< 0.0001	< 0.0001
T2DM + 25% GAG vs. Negative control	5	0.6156	0.0441	> 0.9999	0.6827	< 0.0001	< 0.0001	< 0.0001	> 0.9999	0.0001	0.0078	0.8803	< 0.0001	< 0.0001
T2DM + 50% GAG vs. Normal + 50% GAG	5	> 0.9999	0.9826	0.9995	0.0829	< 0.0001	0.3689	< 0.0001	0.0634	0.1228	0.0179	0.018	< 0.0001	< 0.0001
T2DM + 50% GAG vs. Negative control	5	0.4600	0.8633	0.6086	0.3361	< 0.0001	< 0.0001	< 0.0001	0.0027	0.9862	> 0.9999	0.0004	< 0.0001	< 0.0001
Normal + 50% GAG vs. Negative control	5	0.4704	0.4739	0.4732	0.9717	< 0.0001	< 0.0001	< 0.0001	< 0.0001	0.3432	0.0029	< 0.0001	< 0.0001	0.803

KEY: N: number of rats per group; MDA: malondialdehyde; GPx: glutathione peroxidase; GAG: grain amaranth grain; T2DM: type 2 diabetes mellitus.

## Data Availability

Information used in the study can be found at https://figshare.com/s/0b820f4843ea1276e1f5.
